# Advancing understanding of social prescribing in the Western Pacific region

**DOI:** 10.1016/j.lanwpc.2026.101824

**Published:** 2026-02-27

**Authors:** The Lancet Regional Health – Western Pacific

Social prescribing is an approach in which trusted individuals in clinical and community settings identify patients’ health-related social needs and refer them to community-based activities and services. It can help to reduce social isolation, improve physical and mental health, and enhance overall wellbeing. Current social prescribing programmes are often targeted at specific groups, such as older adults, people with chronic conditions, or those experiencing social isolation.

Although relatively new, social prescribing has gained policy attention and has been implemented in multiple countries across the Western Pacific region. In this issue, regional experts present the Social Prescribing in the Western Pacific region Series, which systematically summarised the existing evidence on social prescribing models and offered perspectives on adapting these approaches to meet diverse health needs. The Series reflects the growing interest in social needs interventions in the Western Pacific region. Despite policy enthusiasm, social prescribing studies in Western Pacific countries are largely at the pilot stage and lack robust evidence regarding delivery models and effectiveness. Limited resources, fragmented referral systems, and a lack of standardised evaluation are the main challenges we face.

The popularity of social prescribing intervention in the Western Pacific region is driven by several factors, including growing recognition of the impact of social determinants on health and wellbeing, the urgent need to reform traditional curative health-care models towards a more holistic and personalised approach, and the region's rich tradition of community-led interventions. Increasing health demands and medical costs due to population ageing and the rising burdens of non-communicable diseases are driving health-care systems to seek more sustainable and effective models. By leveraging existing community resources, social prescribing has the potential to address non-medical causes of disease, enhancing overall health at a relatively low cost.

Although it has been designed as a standardised, replicable delivery model in resource rich settings, social prescribing interventions do not function optimally in dynamic environments with varying resources and capacities for efficient referral pathway and community support. This is particularly challenging in the Western Pacific region, where health-care systems and social support are highly heterogeneous. Michelle Shu Jing Wong and colleagues proposed a stage-sensitive approach using multiple methodologies to assess effectiveness of social prescribing during different implementation stages. The flexibility of this evaluation framework helps to understand social prescribing as a complex service and promote its adaptation in real-world settings.

There is a call for place-based and strengths-based, community-centred approaches that empower communities to improve health by identifying and building upon existing strengths and local resources. Given the region's cultural diversity, Yukiko Honda and colleagues explore the impact of traditional arts and events on mental health and social wellbeing, highlighting the potential psychological and social benefits of cultural approaches. In a community-led social prescribing model called CONNECT Initiative in Lao People's Democratic Republic, unlike being led by health-care workers in high-income countries, the approach leverages the coherent community structure in the country to identify and address social needs by local communities. Although promising, transferring leadership and power to communities could risk shifting responsibility for service provision away from health-care systems and authorities, and onto communities. This might reduce the burden on health-care systems, but excessive reliance on individuals and already constrained, under-funded community services will only exacerbate inequalities, as people in resource-rich settings are better equipped to benefit. People who could benefit most might lack the personal or socioeconomic resources to access medical or community systems.

Social prescribing represents a trend towards community-based care, but its benefits can only be fully realised if we invest in communities first. As a region that historically emphasises individual responsibility for health, social prescribing has the positive impact of changing the mindset that health is not only about an individual's choice on lifestyle but is more fundamentally affected by the society we lived in. Social prescribing is not a quick fix for all determinants of health, but it offers valuable support for a more integrated and holistic health-care system for the region.

